# Temporal Brain Dynamics of Multiple Object Processing: The Flexibility of Individuation

**DOI:** 10.1371/journal.pone.0017453

**Published:** 2011-02-28

**Authors:** Veronica Mazza, Alfonso Caramazza

**Affiliations:** 1 Center for Mind/Brain Sciences (CIMeC), University of Trento, Trento, Italy; 2 Department of Cognitive Sciences and Education, University of Trento, Trento, Italy; 3 Department of Psychology, Harvard University, Cambridge, Massachusetts, United States of America; University of Minnesota, United States of America

## Abstract

The ability to process concurrently multiple visual objects is fundamental for a coherent perception of the world. A core component of this ability is the simultaneous individuation of multiple objects. Many studies have addressed the mechanism of object individuation but it remains unknown whether the visual system mandatorily individuates all relevant elements in the visual field, or whether object indexing depends on task demands. We used a neural measure of visual selection, the N2pc component, to evaluate the flexibility of multiple object individuation. In three ERP experiments, participants saw a variable number of target elements among homogenous distracters and performed either an enumeration task (Experiment 1) or a detection task, reporting whether at least one (Experiment 2) or a specified number of target elements (Experiment 3) was present. While in the enumeration task the N2pc response increased as a function of the number of targets, no such modulation was found in Experiment 2, indicating that individuation of multiple targets is not mandatory. However, a modulation of the N2pc similar to the enumeration task was visible in Experiment 3, further highlighting that object individuation is a flexible mechanism that binds indexes to object properties and locations as needed for further object processing.

## Introduction

Selecting multiple visual objects is a fundamental activity for a coherent perception of the world, and accordingly has been shown to be present in humans of all ages and in animals (e.g., [1]–[3]). For this reason, multiple object processing is a crucial topic in several areas of cognitive neuroscience, such as attention, object recognition and number representation (for a review, see [4]–[8]). By means of Event-Related Potential (ERP) recordings the present study sought to provide converging evidence for a better understanding of the nature of multiple object processing. More specifically, we wanted to assess whether the visual system mandatorily tags all relevant elements or whether multiple object selection is task dependent, being sensitive to the perceptual/cognitive operations required for the execution of a task.

Various models of vision [7], [9]–[12] propose at least two separate mechanisms in object analysis. One mechanism sets up temporary representations of objects, providing a coarse representation of their properties and allowing the visual system to individuate each element as being separate from others. The other mechanism encodes the objects in greater detail, thus leading to identification. How the first stage of “object individuation” operates is not fully understood, although the prevalent hypothesis is that it indexes the relevant elements mainly in a stimulus-driven fashion (e.g., [11], [13], [14]). Thus, one may predict that when the relevant items are perceptually salient, either because they are the only elements in the visual field, or because they possess unique features relative to the other elements (distracters), the individuation mechanism will operate in a way that is determined by target numerosity. In line with this prediction, recent fMRI studies (e.g., [15], [16]) found that activity in a specific parietal area (the inferior intraparietal sulcus, IPS) increases linearly as a function of the number of relevant objects in the visual field, regardless of their complexity. In their neural object-file theory, Xu and Chun [8] interpret this result as evidence that individuation, as reflected by the activity in the inferior IPS, is a mechanism that codes for target numerosity regardless of encoding demands. However, task requirements were not manipulated in their studies. Therefore, whether the visual system can flexibly allocate resources for multiple object individuation is still unclear. Moreover, given the poor temporal resolution of the fMRI technique, these data do not provide information on the temporal brain dynamics involved in multiple object processing.

Recent ERP studies have uncovered the existence of two temporally separated brain activations during the execution of visual attention tasks in which a lateralized target is presented together with some distracters [17]–[21]. While a first lateralized response at posterior electrode sites (N2pc, 180–300 ms; [22], [23]) is always elicited whenever a relevant object is presented in the visual field, a second sustained lateralized activity (Contralateral Delayed Activity, CDA, 350–600 ms; [24]; also called SPCN [19]) occurs only when the task requires the relevant object to be encoded in greater detail. Based on these and other findings (e.g., [25]), we assume that the N2pc is the most likely ERP correlate of the individuation mechanism, while the later sustained activity is related to the cognitive operations involved in object identification and elaboration. However, these ERP findings have all concerned the presentation of a single relevant element (together with various distracters) in the visual display. Thus, the temporal dynamics of multiple object individuation have remained largely unexplored.

A recent ERP study on multiple object tracking [26] showed that the amplitudes of both the N2pc and the CDA were modulated by the number of elements that were tracked. Specifically, both the N2pc and the CDA increased as a function of target numerosity, presumably reflecting the individuation of the target elements and continuous updating of the representation of the individuated objects, respectively. In additional support of the interpretation of the N2pc as reflecting object individuation, N2pc amplitudes reached an asymptote between 3 and 5 tracked elements, indicating a capacity limit similar to the one proposed by models of individuation (e.g., [11]). However, since task requirements were not manipulated, Drew and Vogel's [26] study does not address whether individuation operates in a mandatory fashion determined only by target numerosity, or whether its operation over multiple potential targets depends on task demands.

**Figure 1:**
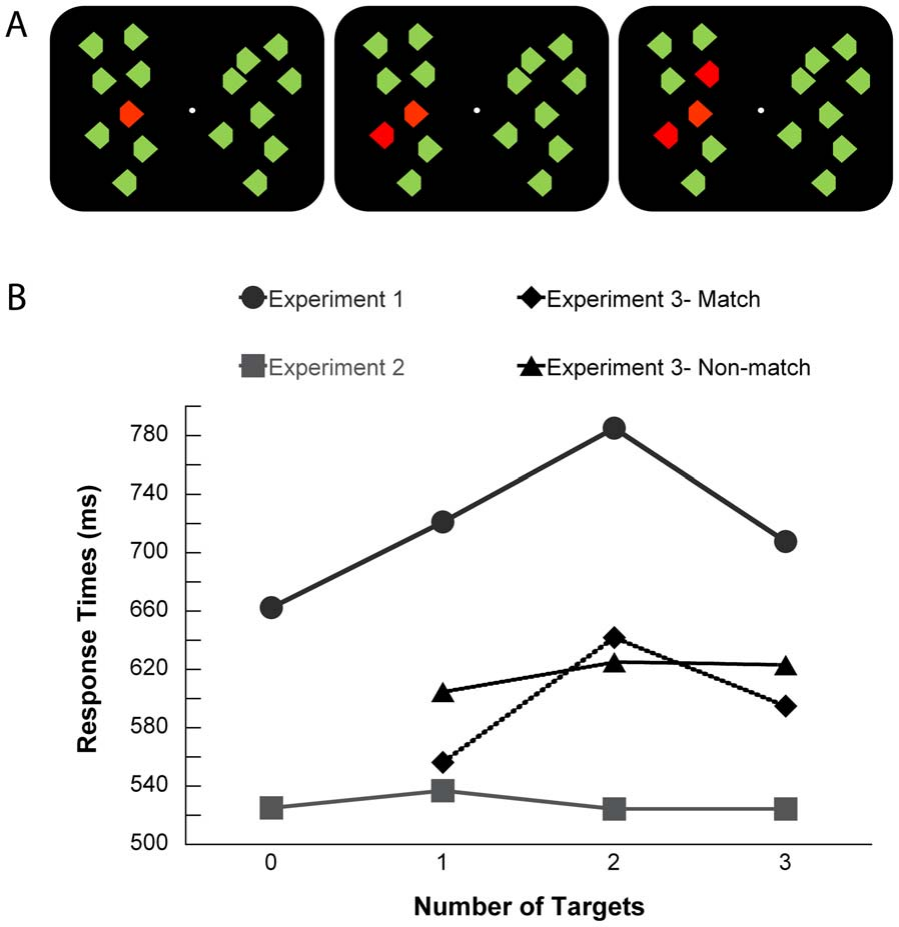
Stimuli and behavioral results. (**A**) Example of trials with one (left), two (middle) and three (right) targets. (**B**) Response times (milliseconds) of all the experiments show an anchoring effect for the extreme target numerosities in Experiment 1 (enumeration) and in the match condition of Experiment 3.

In the first two ERP experiments of the present study participants saw a variable number (zero, one, two or three) of uniquely colored elements presented together with various distracters (see example displays in Figure 1a). The use of distracters served two purposes. First, by creating a cluttered scene it can help accentuate the role of individuation processes in distinguishing and selecting the task-relevant objects. Second, we wanted to have a context similar to the one used in Drew and Vogel's [26] study, as well as in most of the previous studies on the N2pc component (e.g., [23]).

The experiments were identical in terms of the physical parameters being manipulated but varied in the task requirements: counting the number of elements, in Experiment 1, versus reporting whether a target was present independently of their number, in Experiment 2. In order to perform an enumeration task, the visual system has to isolate the elements to be counted both from distracters and from each other [27]. Thus, the individuation stage during the execution of this task should be affected by the number of elements to be counted. For this reason in Experiment 1 we predicted that, as in Drew and Vogel's [26] study, the N2pc would increase as a function of target numerosity. What happens during a simple present/absent judgment (Experiment 2)? One possibility is that no individuation takes place at all, as the system may only need to accumulate evidence for the relevant color feature (e.g., redness). If such were the case, no N2pc should occur for any target numerosity. Alternatively, the visual system might rely on the individuation of only one target (or the grouped set of targets) relative to distracters. In this case, we would expect to find an N2pc but no modulation across target numerosity. Finally, if the visual system mandatorily individuates all the elements of the target set, regardless of whether encoding of multiple object locations is useful for the task at hand, we would expect to find an N2pc, modulated by target numerosity as in Experiment 1 (and as in [26]).

Also based on previous findings, showing that the CDA is modulated by the degree or depth of processing of the relevant objects (e.g., [20], [28]), we expected a CDA for the enumeration but not the detection task. This expectation is based on the plausible assumption that since the enumeration task requires the assignment of a numerical value to the target sets, a detailed representation of the individuated elements would have to be computed, resulting in a modulation of the CDA as a function of target elements. By contrast, in the detection task, target properties do not have to be encoded in detail and therefore no CDA is expected.

Experiment 3 was designed to further assess the hypothesis that individuation is a flexible process that is sensitive to the perceptual/cognitive operations required for the execution of a task. It is important to note that the tasks used in Experiment 1 and Experiment 2 differ in aspects that are not strictly related to individuation. For instance, the numbers of response alternatives required by the two tasks are different (2 for detection, 4 for enumeration). Thus, a difference in the neural activity between these two tasks could in principle be accounted for by response-selection factors that are not intrinsically related to individuation (e.g., [29]).

For this reason, and similarly to the first two experiments, in Experiment 3 we presented a variable number (1, 2, 3) of elements with a unique color together with some distracters. As in Experiment 2, we asked participants to provide a present/absent judgment (two response alternatives), but differently from Experiment 2, for each block a specific target numerosity was designated and a given stimulus could either have the same number (match) or a different number (non-match) of the target-relevant objects. We predicted that in this context the visual system needs to isolate the target-relevant elements in order to reach a decision. Therefore, similarly to Experiment 1, the N2pc should be modulated by the number of target-relevant elements.

## Materials and Methods

### Participants

Thirty-six healthy right-handed paid volunteers (Experiment 1: 8 participants, all females, mean age 19.5 years; Experiment 2: 12 participants, 8 females, mean age 21 years; Experiment 3: 16 participants, 12 females, mean age 22 years) participated in the experiments, after providing written consent. The experiments were conducted following the guidelines laid down in the Helsinki Declaration and were approved by the local ethics committee (Comitato Etico per la Sperimentazione con l'Essere Umano, University of Trento).

### Stimuli and procedure

Stimuli consisted of equiluminant red and green diamonds (17 cd/m^2^) presented on a black background (1 cd/m^2^). Each diamond (0.6°×0.8°) had a 0.4° corner trimmed on the left or right side (see Figure 1a). On each trial, the display contained a total of 16 diamonds, equally distributed to the left and right side of the fixation circle (0.2°). The diamonds were located within a 10 (columns, 11.4°)×8 (rows, 8.6°) matrix. On 1/4 of the trials in Experiment 1 and on half of the trials in Experiment 2, all diamonds had the same color (zero-target condition). On the other trials, one, two or three diamonds (the targets) had a unique color (either red or green) and appeared with equal probability and in random order to the left or right of fixation, but never in the two columns of the matrix closest to fixation. In Experiment 3, one, two or three diamonds with a unique color were presented on each trial. The color of the elements was counterbalanced across participants. Each visual display was presented for 150 ms.

In Experiment 1, participants reported as fast as possible the number of targets presented on each trial by pressing one of four keys with their index or middle fingers of both hands. In Experiment 2, participants indicated the presence (Yes/No) of at least one target element by pressing one of two keys on a computer keyboard with the index fingers of their left or right hand. In Experiment 3 participants indicated the presence (Yes/No) of a specific target numerosity that was designated prior to the start of each block of trials, by pressing one of two keys with the index fingers of both hands. In all experiments response assignment was counterbalanced across participants. Maximum time for responding was 1500 ms. The inter-trial interval was 1500 ms. In Experiments 1 and 2 participants performed ten experimental blocks of 120 trials per block (Experiment 1: 30 zero-target, 30 one-target, 30 two-target and 30 three-target trials; Experiment 2: 60 zero-target, 20 one-target, 20 two-target and 20 three-target trials). In Experiment 3 six blocks of 208 trials per block (104 trials with the target numerosity for a specific block, and 52 trials for each of the other two numbers of elements with a unique color) were delivered.

### EEG recording and data analysis

EEG was recorded from 25 electrodes (including PO7, PO8, O1 and O2) and from a left earlobe electrode, with a right-earlobe reference (bandpass filter: 0.01–40 Hz, A/D rate: 1000 Hz), and then re-referenced offline to the average of the left and right earlobe sites. Horizontal EOG (HEOG) was recorded by means of two electrodes positioned on the outer canthii of both eyes. Trials with horizontal eye movements (HEOG exceeding ±30 µV), eye blinks, head movements, and other artifacts (any electrode exceeding ±80 µV) were excluded.

Averages for correct responses were computed relative to the 100 ms interval preceding the display onset, separately for each condition. Statistical analyses on target-present trials were conducted on mean difference amplitudes obtained by subtracting ERP waveforms at ipsilateral posterior electrodes (e.g., PO7 for left target location, PO8 for right target location, respectively) from those recorded at contralateral sites (e.g, PO8 for left targets, PO7 for right targets) for the following post-stimulus intervals: N2pc (180–270 ms), CDA (350–600 ms). The factors considered were target numerosity (one, two and three targets) and electrode (PO7/8, O1/2). In Experiment 3, the factor match/non-match was also considered. When appropriate, Greenhouse-Geisser correction for sphericity violations was applied, and only the correct p values are reported.

## Results

### Experiment 1

#### Behavioral performance

An ANOVA on RTs for correct responses between 200 and 1500 ms (factor: target numerosity, zero, one, two and three) showed a significant main effect, *F*(3, 21) = 26.86, *p*<.001. Follow-up analyses (*t*-tests) revealed that participants were fastest on trials with zero targets (*M* = 662 ms) than with any other target numerosity, all *p*s<.02. Participants were also faster on trials with one (*M* = 721 ms) or three targets (*M* = 708 ms) relative to two-target trials (*M* = 785 ms), both *p*s<.005 (Figure 1b). No significant difference emerged between trials with one and three targets, p = .32.

Participants' accuracy was high overall (more than 91% of correct responses) and, in line with the RT pattern, greater on zero-target trials (*M* = 98%) relative to one (*M* = 95%) or two-target trials (*M* = 91%), both *p*s<.012, but not on three-target trials (*M* = 97%), *p* = .142. Moreover, accuracy was greater on trials with three targets relative to one- and two-target trials, both *p*s<.04. This pattern of results reveals the beneficial effect of “anchor” positions.

#### ERP results

**Figure 2:**
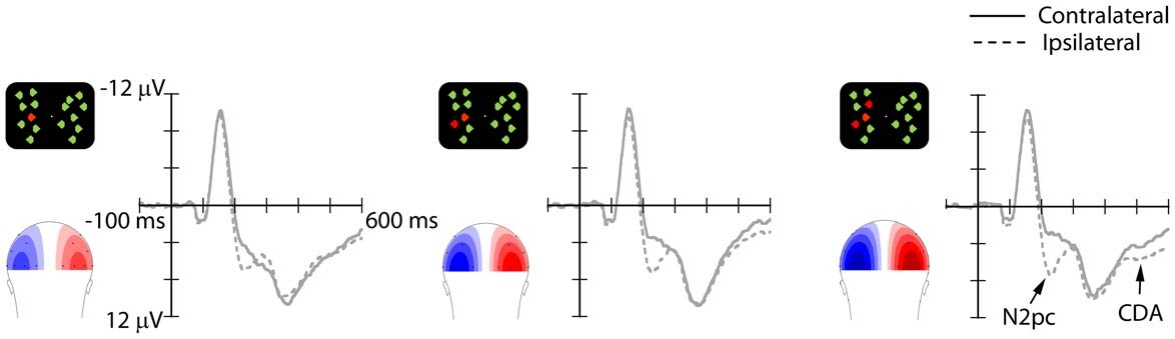
Grand-average ERP waveforms obtained in Experiment 1 in the 600 ms post-stimulus interval at posterior electrodes PO7/PO8 contralateral (solid lines) and ipsilateral (dashed lines) to the target hemifield, as a function of target numerosity (left column: one-target condition; middle column: two-target condition; right column: three-target condition). Topographical ERP scalp distribution maps of the N2pc (180–270 ms) are shown for each condition, obtained by computing the contralateral minus ipsilateral difference activity, and mirrored across the midline. The scale was optimized for each experiment (Experiment 1: ±4 µV). Both the N2pc and CDA increased as a function of target numerosity.

The N2pc was clearly modulated by the number of targets presented in the visual field, being larger for the larger target numerosities (Figure 2). In addition, the N2pc was followed by a CDA showing the same modulation, with larger amplitudes for larger target numerosities. Statistical analyses confirmed these observations.

The ANOVA in the N2pc time range (180–270 ms) showed significant effects of target numerosity, *F*(2, 14) = 22.5, *p*<.001, and electrode, *F*(1, 7) = 12.4, *p* = .01, as well as of their interaction, *F*(2, 14) = 10.1, *p*<.002. However, follow-up comparisons (t-tests) revealed that at both electrode locations the N2pc amplitude was larger for three targets than for one or two targets (O1/2: both *p*s<.001; PO7/8: both *p*s<.005), and for two targets than for one target, (O1/2: p<.005; PO7/8: *p*<.005).

The same pattern was found in the ANOVA on the CDA (350–600 ms), with significant effects of target numerosity, *F*(2, 14) = 24.9, *p*<.001, and of target numerosity x electrode, *F*(2, 14) = 13.4, *p*<.001. Pairwise comparisons confirmed that, at both electrode locations, amplitudes were greater for three targets than for one (O1/2: p<.002; PO7/8: *p*<.002) and two (O1/2: *p* = .03; PO7/8: *p* = .05), and for two targets relative to one target (O1/2: *p*<.001; PO7/8: *p*<.001).

### Experiment 2

#### Behavioral performance

Following the same criteria as in Experiment 1, the ANOVA on RTs showed a significant main effect, *F*(3, 33) = 3.3, *p* = .05. Participants were faster on trials with two (*M* = 524 ms) or three (*M* = 524 ms) targets relative to one-target trials (*M* = 537 ms), *t*(11) = 4.0, *p*<.002 and *t*(11) = 2.3, *p* = .039, respectively. They were also slightly faster in the zero-target condition than in the one-target condition, *t*(11) = 2, *p* = .07 (Figure 1b).

No significant effect emerged from the ANOVA on percentage of correct responses (more than 97% in each condition), *p* = .1.

#### ERP results

**Figure 3:**
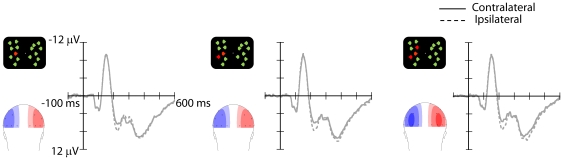
Grand-average ERP waveforms and topographical scalp distribution maps (amplitude scale: ±3 µV) of Experiment 2, obtained as in Experiment 1, showed no modulation of the N2pc and CDA as function of target numerosity.

Two aspects of the present results differed markedly from those of Experiment 1 (see Figure 3). First, an N2pc was clearly elicited in all three conditions, but was not modulated by the number of targets presented in the visual display. No significant main effect of target numerosity or interaction emerged, both *p*s>.2. In addition, no CDA emerged from the analysis in the subsequent time range (350–600 ms), all *p*s>.12.

### Experiment 3

#### Behavioral performance

An ANOVA on RTs for correct responses between 200 and 1500 ms (factors: numerosity – one, two and three; match/non-match) showed a significant main effect of numerosity, *F*(2, 30) = 49, *p*<.001 and a significant numerosity x match/non-match interaction, *F*(2, 30) = 8.4, *p*<.001. In both the match and non-match conditions participants were faster on trials with one (match: *M* = 561; non-match: *M* = 607) than two (match: *M* = 643; non-match: *M* = 629) or three elements (match: *M* = 606; non-match: *M* = 622), all ts>3.2, all ps<.008. In the match condition, they were also faster on three relative to two elements, *t*(15) = 3.1, *p* = .008 (Figure 1b). This pattern of results reveals that the effect of “anchor” positions seen in Experiment 1 is visible even when participants do not have to explicitly report the number of target-relevant elements.

An ANOVA on percentage of correct responses revealed a main effect of numerosity, *F*(2, 30) = 20.96, *p*<.001, and match/non-match, *F*(1, 15) = 5.4, *p* = .03, as well as of their interaction, *F*(2, 30) = 3.97, p = .05. In the match condition, participants' accuracy was greater on one-target trials (*M* = 96%) relative to two-target trials (*M* = 94%), *t*(15) = 3.8, *p* = .002, but not to three-target trial (*M* = 96%), *p* = .06. Two- and three-target trials also differed from each other, *t*(15) = 2.6, *p* = .02. In the non-match condition, participants were more accurate with three (*M* = 98%) relative to one (*M* = 97%) or two target-relevant elements (*M* = 94%), all *p*s<.05.

#### ERP results

**Figure 4:**
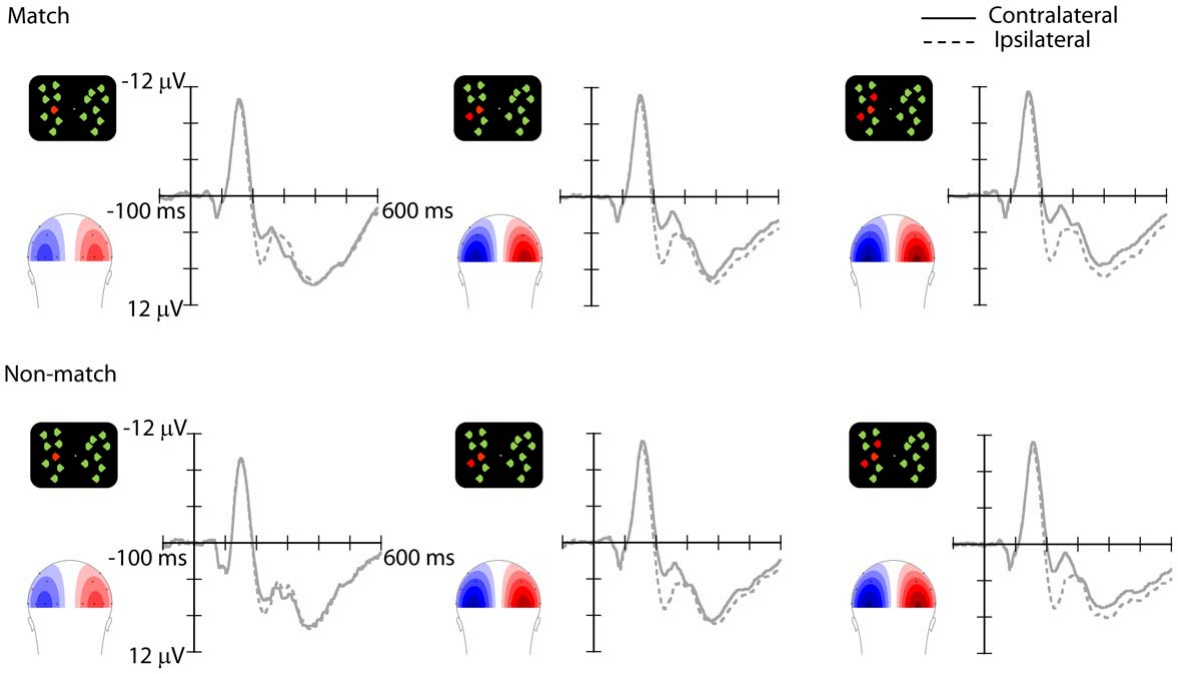
Grand-average ERP waveforms and topographical scalp distribution maps (amplitude scale: ±4 µV) of Experiment 3, obtained as in Experiment 1, showed an increase in the N2pc and CDA amplitudes as a function of target numerosity for both the match and non-match conditions.

**Figure 5:**
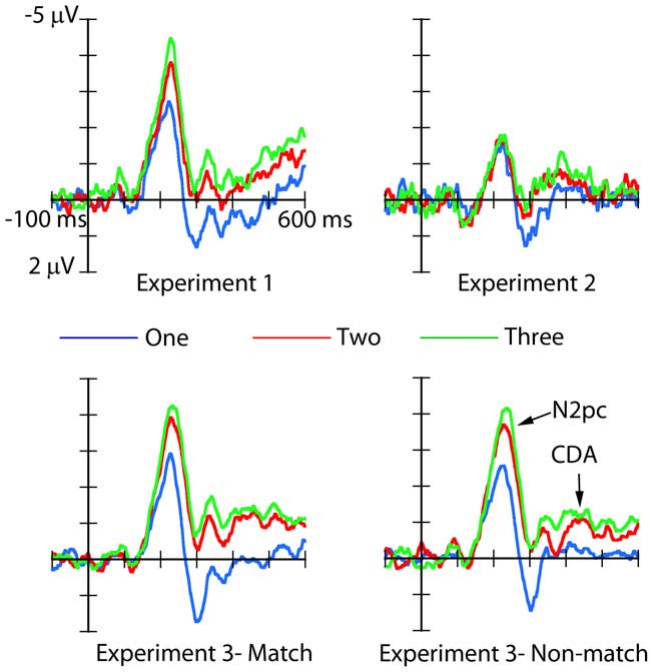
The grand-average difference waveforms obtained by subtracting the ipsilateral activations from contralateral activations at posterior sites PO7 and PO8 show an increase of the N2pc amplitudes as a function of target numerosity only for Experiment 1 (top left) and Experiment 3 (bottom), but not for Experiment 2 (top right).

As in Experiment 1, both the N2pc and CDA were modulated by the number of targets presented in the visual field. (Figure 4; see also Figure 5 where ERP differential waveforms are shown for all the experiments). Interestingly, this was evident for both match and non-match target-relevant numerosities.

The ANOVA in the N2pc time range (180–270 ms) revealed significant effects of numerosity, *F*(2, 30) = 51.54, *p*<.001, and electrode, *F*(1, 15) = 18.5, *p* = .001, as well as of their interaction, *F*(2, 30) = 14.6, *p*<.001. Neither the effect of match/non-match nor any interaction involving this factor was significant, all ps>.4. Follow-up comparisons (t-tests) separately for each electrode site revealed, as in Experiment 1, that the N2pc amplitude was larger for three targets than for one or two targets (O1/2: both *p*s<.001; PO7/8: both *p*s<.02), and for two targets than for one target, (O1/2: p<.001; PO7/8: *p*<.001).

The same pattern was found in the ANOVA on the CDA (350–600 ms), with significant effects of numerosity, *F*(2, 30) = 17.3, *p*<.001, and of target numerosity x electrode, *F*(2, 30) = 11.4, *p*<.001, with pairwise comparisons confirming that, at both electrode locations, amplitudes were greater for three targets than for one or two targets (O1/2: ps<.02; PO7/8: *ps*<.03) and for two targets relative to one target (O1/2: *p*<.001; PO7/8: *p* = .001). A significant match/non-match x numerosity also emerged, *F*(2, 30) = 4.6, *p* = .02. However, follow up comparisons revealed that the CDA was larger for the larger target-relevant numerosities in both the match (all ps<.02) and non-match (all ps≤.05) conditions.

### General discussion

Previous work has shown that the visual system can select multiple items simultaneously [1], [11]. However, the nature of this process is not fully understood. In the present study we tested two hypotheses: a) that selection of multiple objects occurs mainly in a bottom-up fashion: This hypothesis implies that the visual system mandatorily individuates all the potentially relevant elements, regardless of whether this is useful for the task at hand; b) that individuation of potentially relevant items is a flexible mechanism that operates under top-down control, individuating multiple objects only if needed.

The results of the present study clearly favor the second hypothesis. In our data the impact of target numerosity on the N2pc was strongly modulated by task demands: the N2pc increased as a function of the number of targets in the enumeration task (Experiment 1) but not in the detection task (Experiment 2). The occurrence of the N2pc in Experiment 2 implies that target individuation also occurs in a simple detection task (see also [30], [28]). However, the fact that the amplitude of the N2pc did not vary as a function of target numerosity indicates that individuation is either limited to a single element or occurs through a grouping of all the target elements [31], [32]. While future work will need to disentangle these two possibilities, the present results indicate for the first time that the visual system individuates multiple relevant elements in the visual field as a function of task requirements.

Importantly, results of Experiment 3 allowed us to rule out alternative explanations of the different patterns found in the first two experiments in terms of task difficulty related to the number of response alternatives. First, as in Experiment 1 but different from Experiment 2 the N2pc amplitudes increased as a function of target-relevant numerosities for the match condition, even though the type of judgment (present/absent response) was identical to the detection task of Experiment 2. Second, the same modulation of target-relevant numerosities was visible when the non-match trials were considered, indicating that this modulation is uncorrelated with the specific status (i.e., match versus non-match) of the target-relevant elements. These results further support the hypothesis that the visual system selects potentially relevant items as a function of the demands imposed by the task.

In line with other studies showing that the amplitude of the N2pc does not always correlate with the behavioral measures used to assess performance in a particular task (e.g., [17], [20], [21], [26], [28], [33], [34]), in the present experiments we did not find a correlation between RT and N2pc data. Together with the findings from Experiment 3, this further confirms that the modulations of the N2pc seen in the present experiments cannot be accounted for by an overall effect of task difficulty as seen from RTs. For instance, an account based on task difficulty per se would predict, contrary to what was observed, a reduction of the N2pc for the three-target condition in Experiments 1 and 3, and for the larger target sets in Experiment 2, since RTs were faster relative to the one-target condition. More generally, this aspect makes the N2pc a valuable index to test models of vision because it can isolate effects that may not be visible from chronometric data, which reflect the overall product of several and sometimes opposing effects ensuing at different stages of processing.

According to some models of visual object analysis (e.g., [8]) individuation is the mechanism through which an object is distinguished from the others mainly on the basis of spatial location. In addition, this mechanism can operate simultaneously on a small set of objects (approximately four), leading to a rough representation of their properties. The lateralized nature of the N2pc indicates that the visual hemifield where the potentially relevant stimuli occur is processed differently from the other hemifield, thus implying that (at least coarsely) the location(s) of these objects play an important role for the mechanism reflected by this ERP response. For this reason, and given the modulation of its amplitude as a function of target number in the enumeration task, we propose that the N2pc reflects the component of individuation that binds indexes to properties and locations in order to make them available for further cognitive operations. Thus, the present data extend the previous work by Drew and Vogel [26] by showing that the neural modulations found in that study are not restricted to multiple object tracking but are common across different types of tasks in which the visual system needs to isolate the potentially relevant items.

The fact that, differently from Experiments 1 and 3, the N2pc is not modulated by the number of targets in a typical detection task (Experiment 2) indicates that this component can be controlled by top-down factors. While being the first demonstration of the flexibility of multiple object individuation, this result is complementary to two sets of experimental findings. First, it is in line with recent ERP studies on contingent attention capture showing that a salient distracter elicits an N2pc only if it possesses task-relevant features (e.g., [35], [36]). Second, it converges with findings on single-target processing showing an increase in the amplitude of this neural response when the task requires that the relevant item is kept separated from the distracter elements, such as when the relevant item needs to be localized or identified, as opposed to when the task simply requires to detect its presence [28], [30]. Interestingly, these latter findings point out an aspect that has not fully been considered yet, namely the role that distracters may have during individuation of single or multiple targets.

Two related aspects are worth noting. First, not all previous studies have found modulations of the N2pc as a function of object numerosity (e.g., [37]–[40]), despite presumably requiring the individuation of the relevant elements as in our Experiments 1 and 3. For instance, a recent study on visual working memory [37] failed to find a modulation of the N2pc when two versus four targets were presented for a delayed match-to-sample judgment. While differences in the various paradigms used in those studies do not allow for a unique explanation of the discrepancy between those findings and the present ones, a common aspect to the previous studies is the fact that the targets were presented in isolation, being the only elements presented in a specific (cued) hemifield. By contrast, in the present study, as well as in Drew and Vogel's study [26], the potentially relevant elements were presented in a cluttered context, being intermingled with distracters. We speculate that this factor may potentiate the effect of target numerosity on the N2pc amplitudes, as seen in Experiment 1 and Experiment 3 (see also [26]), by accentuating the processing difficulty in distinguishing and keeping the relevant items separated. An alternative explanation of the present results is not strictly related to the number of objects per se, but rather to a difference in the amount of area occupied by the relevant items, which increases as the number of targets increase. While we cannot directly rule out this account, and acknowledging the possibility that the spatial extent of the area in which the targets are presented may have some effects on the N2pc in an enumeration task, previous work [26] has found no effect of area on both the N2pc and CDA, suggesting that the spatial extent of the target area cannot be uniquely responsible for the present effects.

Second, the fact that only a small number of target elements was used in the present study raises the question of whether the N2pc reaches an asymptote for quantities larger than four-five elements, as predicted by previous models on individuation [10]–[12]. In addition to the findings by Drew and Vogel [26], which provide first evidence that the N2pc does not increase between 3 and 5 items, unpublished research (Pagano & Mazza, submitted) on quantity estimation with an extended range of numerosities (i.e., 1–7 elements) has found an N2pc plateau at about five elements. Thus, there is growing evidence indicating that the N2pc satisfies the “limit” criterion of individuation.

The second main finding of this study concerns the modulation of the CDA in Experiments 1 and 3 but not in the detection task of Experiment 2, indicating that this neural response mainly occurs when a representation of the potentially relevant item(s) needs to be maintained active for subsequent cognitive operations (see also [20], [28]). Since enumerating requires the assignment of a numerical value to each target set, it is plausible to assume that more resources had to be allocated for the maintenance of the representation of multiple target-relevant elements relative to a single target-relevant element. In line with this interpretation, the CDA increased in amplitude with increasing number of targets in Experiment 1. Similarly, in Experiment 3, where the target-relevant elements had to be assigned a specific numerical value in order to determine whether they matched the specified target number, the CDA amplitudes increased with larger numerosities in both the match and non-match conditions.

While the brain areas underlying the CDA have not been fully identified, recent MEG and TMS studies indicate a strong correlation between the N2pc and the activity in posterior parietal areas, together with a contribution of occipito-temporal areas [41]–[43]. Thus, the present data converge with several fMRI studies showing that neurons in posterior parietal areas are tuned for quantities (e.g., [44], [45]), and more broadly with the crucial role of posterior parietal areas in the coding of magnitude across different dimensions, such as number, space and time [46]. The fact that the activity of occipito-temporal areas contributes to the generation of the N2pc may suggest that this neural response codes for both the number and the non-spatial properties of the objects [47], [48].

In conclusion, our data suggest that the (lateralized) N2pc response reflects the functioning of a mechanism that finalizes the setting up of the object files by means of index-to-location (and property) binding. As a result of these operations, a coarse representation of the relevant objects becomes available, allowing the visual system to individuate them in the visual field. Importantly, the present study adds to our understanding of multiple object processing by showing that this index-to-location (and property) binding stage is task dependent and therefore subject to top-down influence.
